# The Synergistic Effect of Electric-Field and Adsorption Enhancement of Amino Acid Carbon Dots Significantly Improves the Detection Sensitivity of SPR Sensors

**DOI:** 10.3390/s25133903

**Published:** 2025-06-23

**Authors:** Jing Ouyang, Xiantong Yu, Mengjie Wang, Longfei Wang, Zhao Li, Chaojun Shi, Hao Li, Yufeng Yuan, Jun Zhou, Min Chang

**Affiliations:** 1 Key Laboratory of Optical Technology and Instrument for Medicine, Ministry of Education, College of Optical-Electrical and Computer Engineering, University of Shanghai for Science and Technology, Shanghai 200093, China; 233350664@st.usst.edu.cn (J.O.); wangmengjie97@hotmail.com (M.W.); wlf422724@163.com (L.W.); lizhao_520@163.com (Z.L.); 231200073@st.usst.edu.cn (C.S.); junzhou@usst.edu.cn (J.Z.); changmin@usst.edu.cn (M.C.); 2 Jiangsu Key Laboratory of Crop Genetics and Physiology/Jiangsu Key Laboratory of Crop Cultivation and Physiology, Agricultural College of Yangzhou University, Yangzhou 225009, China; 3 Jiangsu Co-Innovation Center for Modern Production Technology of Grain Crops, Yangzhou University, Yangzhou 225009, China; 4 Research Institute of Smart Agriculture, Agricultural College of Yangzhou University, Yangzhou 225009, China; 5 School of Electronic Engineering and Intelligentization, Dongguan University of Technology, Dongguan 523808, China; yufengyuan@dgut.edu.cn

**Keywords:** surface plasmon resonance (SPR) sensor, amino acid-derived carbon dots (CDs), adsorption kinetics

## Abstract

Surface plasmon resonance (SPR) detection technology is playing an important role in various fields such as food safety and environmental monitoring due to its excellent stability and reliability. However, there is also a growing demand for higher sensitivity in SPR sensors. Therefore, this work developed an SPR sensor based on the synergistic effect of electric-field enhancement and adsorption enhancement by using amino acid-derived carbon dots (CDs). The results showed that the incorporation of amino acid CDs can generate a maximum electric-field enhancement of up to 6.44 × 10^5^ V/m in the near-field region, which is 312% of that achieved by a bare gold film. And the adsorption kinetics results indicate that the active groups on the surface of amino acid CDs exhibit a notable adsorption enhancement effect for the target molecule (NaCl), with an adsorption capacity 335% higher than that of the bare gold film. This designed SPR sensor demonstrates a detection sensitivity of 167.28 a.u./RIU for NaCl solution, representing a 247.8% improvement compared to an SPR sensor without amino acid CDs under the same conditions. This SPR sensor shows promising potential for applications in biomedical and environmental detection fields.

## 1. Introduction

Surface plasmon resonance (SPR) sensors, as highly advantageous analytical tools, play an indispensable role in numerous fields. In the biomedical domain, the SPR sensors can be used for disease diagnosis and drug screening [1,2]. In environmental monitoring, they enable precise identification of trace pollutants [3]. In food safety, SPR sensors facilitate the detection of harmful substances [4]. Owing to their label-free operation, real-time monitoring capability, and high sensitivity, SPR sensors have become a vital detection method in practical applications [5,6].

Nevertheless, as detection requirements toward lower concentrations and more complex systems, the sensitivity of SPR sensors faces significant challenges [7,8,9,10,11]. To meet these performance demands, researchers are actively exploring various enhancement strategies to improve SPR sensor sensitivity. Common approaches primarily include electric-field enhancement and adsorption enhancement. Electric-field enhancement typically employs nanoparticles or two-dimensional (2D) materials, such as noble metal nanoparticles and graphene. Noble metal nanoparticles leverage the coupling effect between localized surface plasmon resonance (LSPR) and the SPR, which could amplify the nearby electric-field intensity. Meanwhile, the layered stacking of 2D materials with noble metal films facilitates charge transfer between different materials, leading to localized electric-field enhancement. Adsorption enhancement, on the other hand, is achieved by increasing adsorption strength and active sites. Examples include antigen–antibody-specific binding and functionalizing the sensor surface with specific groups to adsorb more target molecules, thereby amplifying detection signals [12,13].

Carbon dots (CDs) are zero-dimensional carbon-based nanomaterials with particle sizes typically below 10 nm. The synthesis of CDs is straightforward and easy to operate. Moreover, CDs possess abundant surface functional groups, such as carboxyl and hydroxyl groups, enabling easy modification with various organic molecules or polymers. This characteristic endows them with significant potential for binding with various target analytes [14,15]. Additionally, CDs exhibit tunable optical properties, with a controllable refractive index range of 1.4–2.2 [16]. Compared to metal quantum dots, CDs demonstrate superior biocompatibility, lower cytotoxicity, and reduced environmental impact. Since their discovery in 2004, CDs have emerged as a research hotspot, showcasing tremendous application potential in materials science, biomedicine, sensing, and other fields [14,17,18,19]. Hou et al. developed an ultrasensitive optical fiber SPR sensor enhanced by an Au-NPs film modified with functionalized CDs for label-free detection of cobalt (II) ions [20]. Farokhnasab et al. found that TiO_2_/CDs composite materials exhibit enhanced antibacterial efficacy [21]. Alavi et al. investigated protein–DNA interactions, confirming that quantum dots can significantly amplify SPR signals [22]. Ahmad et al. demonstrated that NCQD-PVA composite films exhibit superior performance in chlorophyll detection [23].

In this work, we investigated the synergistic effect of electric-field enhancement and adsorption enhancement in SPR sensor design by leveraging the optical properties of amino acid-functionalized carbon dots (CDs), achieving a significant improvement in detection sensitivity. Experimental results demonstrate that this synergistic strategy markedly enhances the sensitivity for NaCl detection. This work provides a novel approach for optimizing SPR sensor performance, offering promising potential to advance more efficient and precise detection applications in life sciences and environmental monitoring.

## 2. Materials and Methods

### 2.1. Reagents

**Figure 1:**
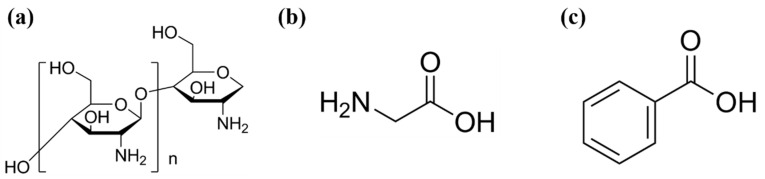
The molecular structures of (**a**) chitosan, (**b**) glycine and (**c**) benzoic acid.

NaCl, chitosan (CS, medium molecular weight), acetic acid (assay ≥ 99.7%), glycine and benzoic acid were purchased from Sigma-Aldrich. Deionized water (18.2 MΩ·cm^−1^) was used throughout the experiment. The chemical structures of CS, glycine and benzoic acid are illustrated in Figure 1.

### 2.2. Synthesis of Carbon Dots

Typically, 1 g of carboxybenzene and 1.25 g of glycine were placed into a Teflon-lined stainless-steel autoclave followed by 25 mL of deionized water and heated at 200 °C for 48 h under static conditions. After cooling to 25 °C, the bright yellow solution was dialyzed using a 500 Da dialysis bag against deionized water to remove the impurities and obtained the CD solution [24,25].

### 2.3. Characterizations

The size, dimension and distribution of the synthesized CDs were characterized using a FEI/Philips Tecnai G2F20 TWIM TEM. The ultraviolet–visible (UV-Vis) spectrum of the amino acid carbon dots was characterized using a GBC UV-Vis spectrophotometer (Cintra2020, Melbourne, Australia). The Raman spectrum of the CDs was characterized using an XploRA Plus microscopic Raman spectrometer (Horiba, Ltd., Kyoto, Japan). The surface morphology of the finalized sensor chip was characterized using a Thermo Apreo 2S HiVac scanning electron microscope (Waltham, MA, USA).

### 2.4. Preparation of Sensing Chip

**Figure 2:**
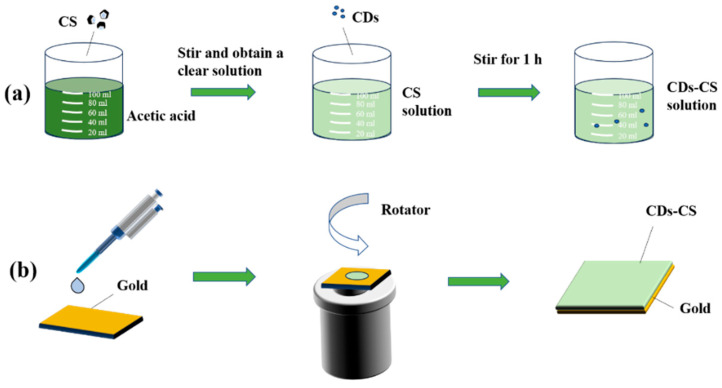
(**a**) Preparation of experimental reagents; (**b**) fabrication of composite sensor chip.

Dissolve 400 mg of CS in 50 mL of 1% acetic acid, stir it thoroughly, and then let it stand overnight at room temperature. The supernatant was taken to obtain the CS solution [26], as shown in Figure 2a. Then, add 100 μL of CDs into 5 mL of the CS solution, and stir for one hour until a uniform CDs-CS solution is formed [27]. At the same time, prepare NaCl solutions with concentrations of 0.1%, 1%, 3%, 6%, 9%, and 15%.

The gold film is manufactured by Suzhou Mixin Semiconductor Co., Ltd. (Suzhou, China) according to our design by using magnetron sputtering technology. First, a 2 nm Cr layer on an SF10 glass substrate with dimensions of 1 mm × 20 mm × 20 mm, and then a 45 nm gold layer is deposited above the Cr layer. The purpose of depositing the Cr layer is to enhance the adhesion of the gold film without affecting the SPR effect. Take 0.5 mL of the above solution and use a spin coater to spin at a speed of 3000 rpm for 60 s to prepare different films on the substrate [27]. As shown in Figure 2b, a silicone microtube is used to introduce the sample solution into the flow channel through an injection pump.

### 2.5. Apparatus

**Figure 3:**
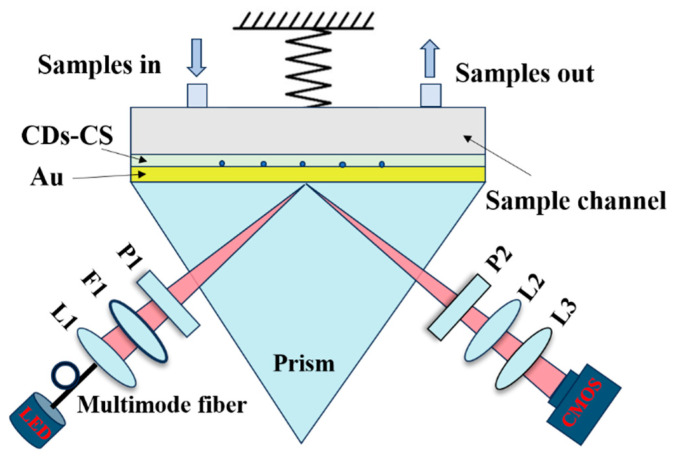
Schematic diagram of the sensing device: L1, L2 and L3: lenses; F1: narrowband filter; P1 and P2: polarizers.

The SPR sensing experiment used a self-made intensity interrogation surface plasmon resonance imaging (SPRi) biosensing system which is based on the Kretschmann structure. The optical setup of this system is as follows: White light is generated by an LED light and introduced into the system through a multimode optical fiber. It is converted into parallel light by a collimating lens and a narrowband filter. The surface plasmon waves excited in the gold–CS–CDs interface by the parallel light are coupled with a prism. A CMOS camera receives the reflected light through an imaging lens group, thereby obtaining a sensing image. The schematic diagram is shown in Figure 3. Subsequently, with the aid of software, the average intensity value of the pixels within the region of interest is obtained to observe the dynamic changes in the intensity of the reflected light caused by the SPR effect in real time and intuitively.

## 3. Results and Discussion

**Figure 4:**
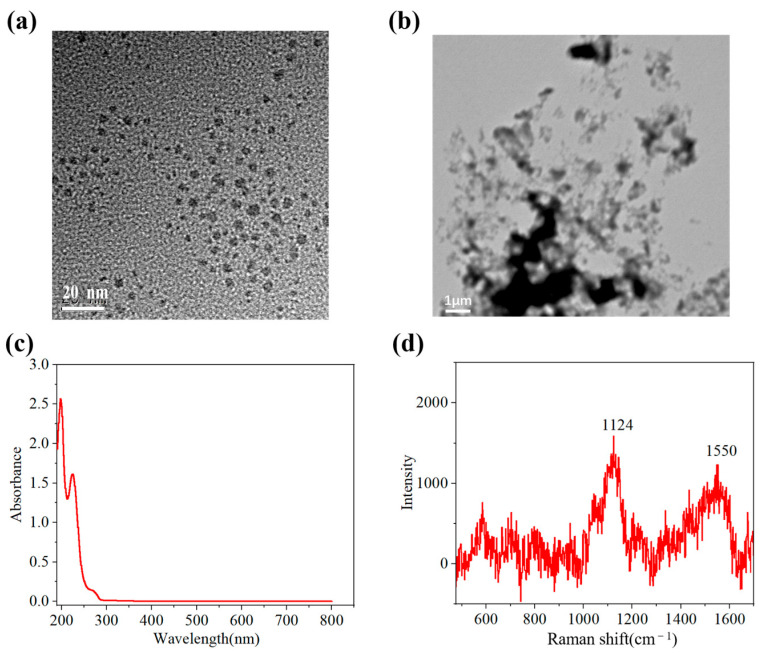
(**a**) The TEM image of CDs. (**b**) The SEM image of Au/CDs-CS ship. (**c**) The UV–Vis absorbance spectra of CDs. (**d**) Raman spectra of CDs.

The morphology, ultraviolet–visible absorption spectrum, and Raman spectrum of the synthesized CDs are shown in Figure 4. As shown in Figure 4a, the CDs exhibited good monodispersity, and their size was approximately 5 nm. The surface morphology of the Au/CDs-CS chip is shown in Figure 4b. The spectrum of the amino acid carbon dots showed three absorption peaks at 197 nm, 224 nm, and 272 nm (Figure 4c). These CDs have a relatively low absorption intensity in the visible light region, indicating that in the SPR working area, the absorption of the CDs will not have a significant impact on the linewidth and reflectivity of the SPR spectrum, thereby ensuring good detection performance of the sensor. Since the pH was 4.14, the amino functional groups played a role in this pH environment. The peaks at the positions of 1124 cm^−1^ corresponded to the C-N bonds formed during the carbonization process of the amino group (-NH_2_) in glycine, respectively (Figure 4d). The peak at 1550 cm^−1^ was close to the G peak of sp^2^ hybridized carbon but lower than the common position of the G peak, which might imply a lower degree of order of the carbon structure or a change in the electronic structure of the carbon lattice due to nitrogen doping.

**Figure 5:**
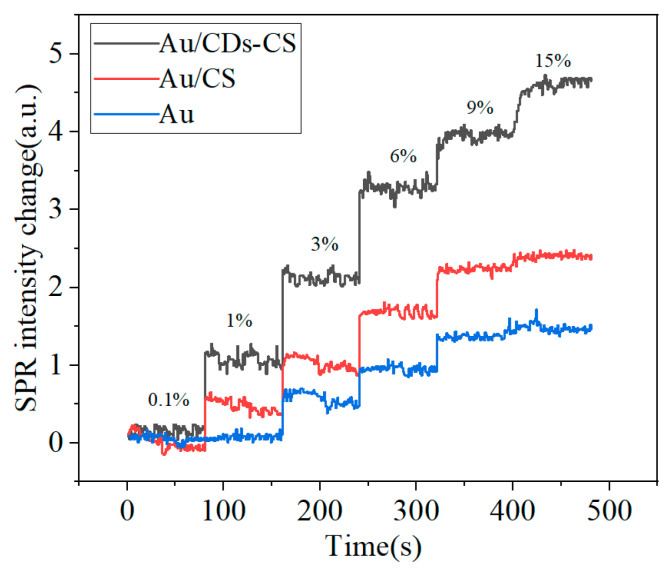
Real-time SPR response of different chips at different NaCl concentrations.

During the experiment, CDs were prepared and mixed with CS, then spin-coated on the surface of the gold film. Meanwhile, a gold film with only CS spin-coated was prepared as a control group. First, deionized water was injected into the microfluidic chamber using a syringe pump to obtain a calibration signal. Subsequently, NaCl solutions of different concentrations were injected into the microfluidic chamber in sequence, and a real-time sensing curve of intensity changing with concentration was obtained (Figure 5). To more intuitively compare the performance of different chips, the calibration signal was set to zero, and the relative intensities corresponding to different concentrations were obtained.

The pKa of CS is approximately 6.5. When the pH of the sample environment is lower than 6.5, the amino groups (-NH_2_) on the CS molecular chain will combine with protons (H^+^) to form positively charged -NH_3^+^_, making the CS positively charged and capable of adsorbing Cl^−^ [28,29,30]. As can be seen from Figure 5, under the condition of the same concentration difference, the sensing chip with CDs added has a stronger response signal, which is significantly better than the CS chip without CDs. This may be because the addition of CDs enhances the electric field at the sensing interface. At the same time, it increases the specific surface area of the chip, improves the dispersibility and stability, and is more conducive to the binding of NaCl molecules to the sites, thereby enhancing the detection signal.

**Figure 6:**
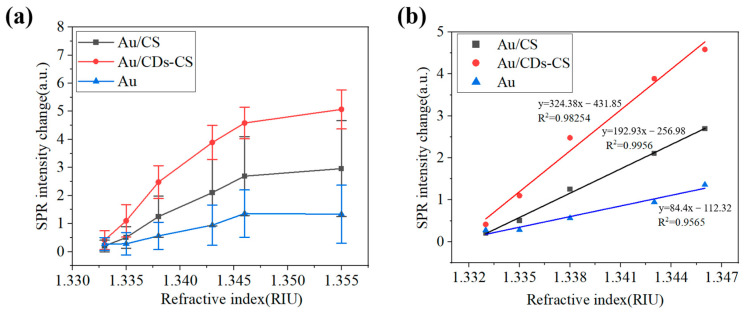
(**a**) Graph of the intensity variation in different chips with the refractive index, and (**b**) linear response.

As can be seen from Figure 6, with the increase in the refractive index, the SPR intensity shows an upward trend. The growth slope of the CDs-CS curve is greater than that of the CS and bare gold film curves, indicating that under the same change in refractive index, the change in SPR intensity of the CDs-CS composite material is more significant. Moreover, at each measurement point, the SPR intensity of CDs-CS is higher than that of CS and the bare gold film. The sensitivity of the two types of chips was calculated according to Equation (1), where RIS represents the ratio of the change in SPR intensity to the change in refractive index, $\Delta I_{SPR}$ represents the change in SPR intensity, and Δn represents the change in the refractive index of the measured medium. The refractive index range of NaCl is 1.333–1.355. According to the calculation results, the sensitivity of CDs-CS is the highest, which is 167.28 a.u./RIU, that of CS is 94.96 a.u./RIU, and that of the bare gold film is 48.09 a.u./RIU. It represents a 247.8% improvement compared to an SPR sensor without amino acid CDs under the same conditions.

(1) $$RIS=\Delta I_{SPR}/\Delta n$$

This article also conducts a study on the stability of the sensor chip. As can be seen from Figure 6a, compared with CS and the bare gold film, the stability of CDs-CS at high concentrations has been significantly improved. In addition, the stability of the bare gold film is better than that of CS. This may be because CS is a high-molecular polymer composed of many glucosamine units connected together. The uneven sites on the surface of the gold film lead to poor stability. After adding CDs, the dispersibility of the surface of the gold film is improved, thus enhancing the detection stability. The calibration curves were shown to verify linear response (Figure 6b). We selected a linear segment with NaCl concentrations ranging from 0.1% to 9% and removed the point with a concentration of 15%.

**Figure 7:**
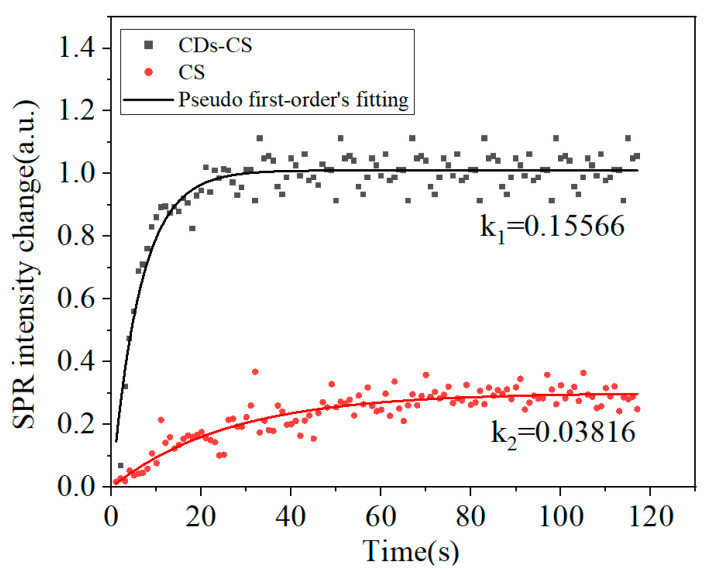
Pseudo-first-order adsorption kinetic models for NaCl-CDs-CS.

Then a pseudo-first-order kinetic adsorption analysis [31] was carried out for the NaCl concentration ranging from 9% to 15% to explore the kinetic process of the interaction between NaCl, CDs, and CS. This interaction is represented by the adsorption rate constant *k*, and the adsorption capacity is represented by the change in intensity, as shown in Equation (2). As can be seen from Figure 7, during the interaction process, the SPR intensity will increase as the NaCl molecules bind to the surface of the sensor and reach a stable state when the adsorption is nearly completed. After reaching equilibrium, the sensor signal remains stable, reflecting the maximum binding capacity of the sensor surface at a given concentration. A larger *k* value indicates a faster adsorption rate, a greater adsorption amount in the same time, and a stronger interaction. The adsorption rate constant *k*_1_ of CDs-CS is 0.15566, and the adsorption amount is 1.01051, while for CS, *k*_2_ = 0.03816, and the adsorption amount is 0.30103, indicating that the CDs-CS composite material has a stronger binding ability to NaCl. Due to the limitations of the response of the bare gold film to low concentrations of NaCl and the weak adsorption effect, it is difficult to obtain accurate data for fitting the adsorption model.

(2) $$\Delta I_{t}=\Delta I_{e}\left[1-\exp\left(-kt\right)\right]$$

**Figure 8:**
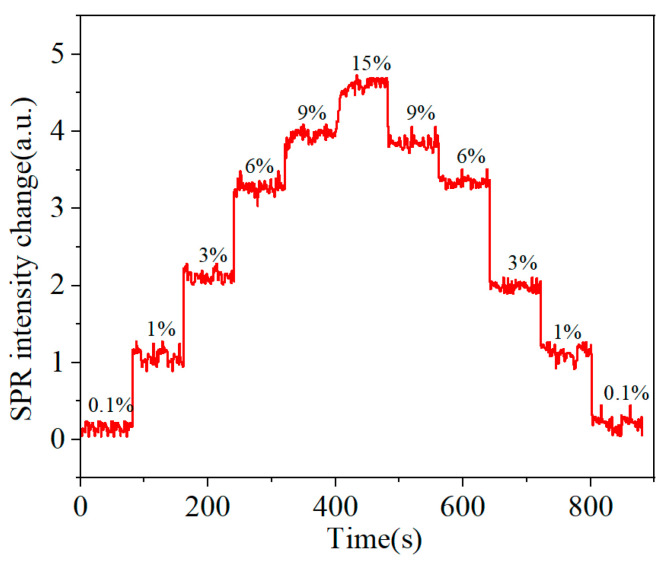
Dynamic response of the CDs-CS chip detection.

After experimentally verifying that the addition of CDs significantly enhances the sensing signal, it is necessary to study its dynamic response and reversibility. During the dynamic detection process, solutions with different refractive indices were continuously injected into the microfluidic chamber and recorded in real time. Figure 8 shows the dynamic response of the NaCl detection. The detection system exhibits good reversibility during the positive and negative cycles of the dynamic refractive index, and the average deviation of the intensity is less than 0.2 a.u., which can be regarded as the error caused by different cycle directions.

**Figure 9:**
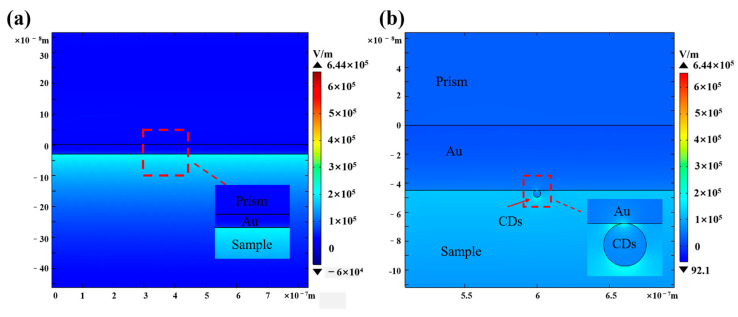
(**a**) The electric field distribution of the Au sensor chip. (**b**) The electric field distribution of the Au/CDs sensor chip.

Finally, we used the finite element method to calculate the electric field distribution at the interface between CDs and the metal (Figure 9). The CDs are 5 nm away from the gold film. The refractive index of CDs was 1.592 + 0.042i, measured by ellipsometer and consistent with literature reports [16]. The electric field of the Au sensor chip is 2.06 × 10^5^ V/m, and the electric field of the Au/CDs is 6.44 × 105 V/m. The simulation results show that the addition of CDs enhances the electric field of the sensor surface, increasing it by 312%, which is consistent with the experimental results.

Table 1 shows the sensitivity of the different SPR sensors using quantum dots on their own or in combination with other materials. From the table, it can be seen that the sensitivity of SPR sensors has been significantly improved after adding quantum dots.

## 4. Conclusions

In conclusion, this work has developed an SPR sensor based on an amino acid CDs-CS film, which achieved a significant improvement in detection sensitivity through synergistic effects. The results have shown that CDs enhance the adsorption capacity of the sensor surface and increase the adsorption amount. Meanwhile, the simulation results indicate that the electric field at the sensing interface is significantly enhanced, confirming the existence of the synergistic effect of CDs, which is the main reason for the improvement in sensitivity. Compared with the sensitivity of the bare gold film, the sensitivity of this sensor reaches 167.28 a.u./RIU, with an increase of 247.8%. From the perspective of practical applications, this synergistic effect of CDs provides a brand-new idea for the design of SPR sensors, which can further expand the application of SPR sensors in different fields by adjusting the preparation process of CDs and optimizing the composite method with other materials.

## Figures and Tables

**Table 1:** Improvement in sensitivity of different SPR sensors after adding quantum dots.

Materials	Target	Sensitivity	Magnification (Ag/Au)	References
Ag/NCDs-PVA	Chlorophyll	1.90 nm/ppm	850%	[23]
Au/CDs	Mn^2+^	6.383 nm/lg(ppb)	-	[32]
Au/CGDs-NCC	Glucose	13.296°/μM	-	[33]
Au/Qdot-StAv	G-quadruplexes(DNA)	75.26 μRIU	494.94%	[22]
Au/CDs-CS	NaCl	167.28 a.u./RIU	247.8%	This work

## Data Availability

The data presented in this study are available on request from the corresponding author.
